# Revealing the diversity of amber source plants from the Early Cretaceous Crato Formation, Brazil

**DOI:** 10.1186/s12862-020-01651-2

**Published:** 2020-08-20

**Authors:** Leyla J. Seyfullah, Emily A. Roberts, Alexander R. Schmidt, Eugenio Ragazzi, Ken B. Anderson, Daniel Rodrigues do Nascimento, Wellington Ferreira da Silva Filho, Lutz Kunzmann

**Affiliations:** 1 Department of Palaeontology, University of Vienna, Althanstraße 14, 1090 Vienna, Austria; 2 School of the Environment, Geography and Geosciences, University of Portsmouth, Burnaby Road, Portsmouth, PO1 3QL UK; 3 Department of Geobiology, University of Göttingen, Goldschmidtstraße 3, 37077 Göttingen, Germany; 4 Department of Pharmaceutical and Pharmacological Sciences, University of Padova, Largo E. Meneghetti 2, 35131 Padova, Italy; 5 School of Earth Systems and Sustainability, Southern Illinois University, Carbondale, IL 62901 USA; 6 Department of Geology, Federal University of Ceará, Fortaleza, CE 60440-554 Brazil; 7 Senckenberg Natural History Collections Dresden, Museum of Mineralogy and Geology, Koenigsbruecker Landstraße 159, 01109 Dresden, Germany

**Keywords:** Amber, Araucariaceae, Cheirolepidaceae, Erdtmanithecales, *Eucommiidites*, Gnetales, In situ pollen, Resin

## Abstract

**Background:**

Amber has been reported from the Early Cretaceous Crato Formation, as isolated clasts or within plant tissues. Undescribed cones of uncertain gymnosperm affinity have also been recovered with amber preserved in situ. Here, we provide multiple lines of evidence to determine the botanical affinity of this enigmatic, conspicuous cone type, and to better understand the diversity of amber-source plants present in the Crato Formation and beyond.

**Results:**

A new taxon of amber-bearing pollen cone *Araripestrobus resinosus* gen. nov. et sp. nov. is described here from complete cones and characteristic disarticulated portions. The best-preserved cone portion has both in situ amber infilling the resin canals inside the preserved microsporophyll tissues and pollen of the *Eucommiidites-*type. This places this genus within the Erdtmanithecales, an incompletely known gymnosperm group from the Mesozoic.

FTIR analysis of the in situ amber indicates a potential araucariacean conifer affinity, although affinity with cupressacean conifers cannot be definitely ruled out. Pyr-GC-MS analysis of the *Araripestrobus resinosus* gen. nov. et sp. nov. in situ fossil resin shows that it is a mature class Ib amber, thought to indicate affinities with araucariacean and cupressacean, but not pinaceous, conifers. This is the first confirmed occurrence of this class of amber in the Crato Formation flora and in South America, except for an archaeological sample from Laguna Guatavita, Colombia.

**Conclusions:**

The combined results of the cones’ novel gross morphology and the analyses of the in situ amber and pollen clearly indicate that the new taxon of resinous gymnosperm pollen cones from the Crato Formation is affiliated with Erdtmanithecales. The cone morphology is very distinct from all known pollen cone types of this extinct plant group. We therefore assume that the plant group that produced *Eucommiidites-*type pollen is much more diverse in habits than previously thought. Moreover, the diversity of potential amber source plants from the Crato Formation is now expanded beyond the Araucariaceae and the Cheirolepidiaceae to include this member of the Erdtmanithecales. Despite dispersed *Eucommiidites* pollen being noted from the Crato Formation, this is the first time macrofossils of Erdtmanithecales have been recognized from the Early Cretaceous of South America.

## Background

Amber (fossilized plant resin) has rarely been reported from South America. The only significant fossiliferous deposit that has been described is from the middle Miocene Pebas Formation of Peru and is thought to be angiosperm in origin [1]. This amber contains inclusions of diverse arthropods, pollen and spores [1]. Small amounts of amber are recorded from the Miocene of the Pirabas Formation near Capanema, Pará, Brazil, and are thought to derive from the angiosperm *Hymenaea* (Leguminosae) [2, 3]. Oligo-Miocene amber from Pecket Mine (Punta Arenas), Chile, may have affinities to podocarpalean conifers [4]. Amber is also present inside fossil conifer (*Agathis*) tissues recovered from the early Eocene Laguna del Hunco flora and the early middle Eocene Río Pichileufú flora, both in northwestern Patagonia, Argentina [5].

There are also undated fossil resins reported from South America. These may be ambers or sub-fossil resins (copals), or even resins; all are angiosperm-derived. The Guayaquil (Ecuador) deposit is thought to have affinities to *Protium* (Burseraceae) and the two Colombian deposits (Gíron and Medellín) are both thought to be derived from *Hymenaea* [2].

Older Early Cretaceous (upper Aptian-lower Albian) South American amber has been reported from Brazil ([6] and references therein). There are three Early Cretaceous ambers from Brazilian locations: Amazonas (Alter do Chão Formation), Araripe (Crato Formation) and Recôncavo (Maracangalha Formation, Caruaçu Member). Using GC-MS, affinities with conifers were suggested for all three ambers including Araucariaceae, Cupressaceae or Cheirolepidiaceae, although in case of the Recôncavo amber, a potential Podocarpaceae origin was additionally inferred [7].

Here, we focus on amber-bearing cone fossils from the Early Cretaceous of the Araripe Basin, Ceará, Brazil. Amber occurs as isolated clasts and preserved within plant fossils [8]. This amber was thought to be derived from araucariacean plants, as both the chemical analysis of isolated amber clasts and the associated macrofossils with in situ (but chemically untested fossil resin) appear to support this [8], although [7] broadened the potential affinities of the amber source plants. The presence of resin canals within the tissues of the conifers *Pseudofrenelopsis* (Cheirolepidiaceae) and *Brachyphyllum obesum* (Araucariaceae) present in the Crato Formation flora has also been shown [9]. This suggests that both these conifer families could potentially be sources for the Crato Formation amber clasts.

We investigated previously undescribed cones with amber preserved to understand their biological affinity, and the diversity of potential amber source plants present in the Early Cretaceous Crato Formation flora. Using a combination of extracting in situ pollen and amber analyses, we uncovered the presence of a further, previously unknown potential source plant of amber in the Crato Formation. The recovered in situ pollen of *Eucommiidites*-type places the new taxon within Erdtmanithecales.

Our study thus contributes to a better understanding of the habit and *bauplan* of the extinct Erdtmanithecales and is likely able to add characters to enlighten its relationships to Gnetales and Bennettitales (BEG (Bennettitales-Erdtmanithecales-Gnetales) group, e.g., [10]) as well as to conifers. Of all the gymnosperm groups possibly implicated in the origin of the angiosperms, the Erdtmanithecales are the least well-known. They are thought, based on the shared presence of chlamydospermous seeds, to possibly have a close relationship with the Bennettitales and Gnetales [10], although this hypothesis remains controversial.

## Methods

Eight cones and cone fragments, one of them with both in situ amber pieces and in situ pollen preserved from the C6 limestone unit of the Crato Formation, Santana Group, northeastern Brazil were investigated. Both the in situ amber and pollen were extracted and analysed to understand the biological affinity of this plant as it had not been previously described.

The specimens are from the following repositories: The Museum für Naturkunde Berlin (specimens prefixed with a MB.Pb. number), University of Portsmouth Collection (specimens prefixed with UOP-PAL-MC), Senckenberg Research Institute and Natural History Museum Frankfurt/M. (specimens prefixed with SM.B), Universidade Estado de Rio de Janeiro (specimen prefixed with UERJ) and Fundação Paleontológica Phoenix, Ceará, Brazil (specimens prefixed with FPH). Complete cone specimens investigated: SM.B 16594; MB.Pb.1997/1350; MB.Pb.2020/0020 (rubber cast). Cone fragment specimens investigated: FPH-83-B; MB.Pb.1997/1246; MB.Pb.1999/1440; SM.B 16599; UOP-PAL-MC0004. Additionally, a resinous specimen of *Brachyphyllum obesum* (UERJ 15-P1) was used to provide in situ amber for the comparison of amber chemistries.

### Geological setting and preservation

The Late Jurassic to Early Cretaceous Araripe Basin, northeastern Brazil is a tectonic depression related to the opening of the South Atlantic (e.g., [11]). Its sedimentary history is linked to the rifting processes and can be broadly subdivided into pre-rift, rift and post-rift tectonic sequences [12]. Recent lithostratigraphic concepts place the post-rift-phase sediments of the Barbalha, Crato, Ipubi and Romualdo Formations into the late Aptian-early Albian Santana Group [13]. The Crato Formation represents mainly lacustrine carbonates and siliciclastic sediments, which are interfingered, at least at its top, by possible fan delta deposits [14]. Six informal limestone units (C1-C6) are distinguished [15], of which C6 at the top of the formation is commercially important and is excavated as “Pedra Cariri” in several quarries between the towns Nova Olinda and Santana do Cariri, Ceará [16]. Fossil biota are therefore almost exclusively recorded from the C6 limestone, which is the case with our material. The exact stratum or strata within the C6 is rarely known because most C6-derived fossils are found by workers in the quarries, who do not exactly record the location of the specimens. This massively hampers floristic and faunal reconstructions of the Crato ecosystem to date. The entity of lacustrine limestone of the Crato Formation has also been referred to as the fossiliferous Nova Olinda Member in other literature (e.g., [17]) and has been sometimes subdivided into two units [18].

The Crato Formation yields an important Gondwanan fossil Konservat Lagerstätte [17], and is particularly considered to be late Aptian to earliest Albian (~ 115 million years old) in age, based on palynological data [18, 19]. This Konservat Lagerstätte is a para-autochthonous to allochthonous assemblage with diverse insects, vertebrates including pterosaurs, and plants. As mentioned above, the Lagerstätte is exclusively described to date from the C6 limestone, a finely laminated rhythmic carbonate deposited in shallow water under mainly hypersaline conditions [17, 20]. Whether the Crato Lake was exclusively a freshwater lake, or temporarily a brackish lagoon from first marine ingressions, was a matter of debate [17, 21] but fully marine taxa are not present. The assemblage constitutes fossil remains of both aquatic and terrestrial biota, with the terrestrial organisms likely either blown in or drifted in by flood events (e.g. [22]) and ephemeral rivers [15, 23].

The plant fossils from the C6 limestone are mostly preserved as iron monosulfide (usually greigite) replacements in the unweathered material, or as goethite (hydrated iron oxide) replacements in weathered material [24]. Coalified (compression) fossils are mainly recorded from the basal “Seven-Cuts”, an informal level of the C6 limestone (pers. comm. from quarry workers to Wellington Ferreira da Silva Filho). Based on the sediment characteristics we infer that at least some study specimens (FPH-83-B; SM.B 16599) originate from the “Branco” Zone (white zone) of the C6 limestone, which is three to four meters above the base of the C6 limestone. The informal zonation of C6 and its terminology are used by the limestone quarry workers in order to localize the excavation position and to distinguish the specific lithological character and quality of the cut stone. Among the 40 taxa of fossil plants, which are validly published to date from the C6 limestone, a pteridophyte [25], cycads [26], diverse conifers [27–29], gnetaleans [22, 30–33] and angiosperms [34–37] have been described, along with isolated pieces of amber [6–8] and charcoalified wood [37].

### Photography

Specimens were photographed with a Canon 60D camera. Light microscopy images were taken using a Carl Zeiss Discovery V8 stereo microscope with a Canon 80D camera attached.

### Collection of amber samples

Amber samples were removed from the cone and *Brachyphyllum* specimen using sharpened needles, then screened and cleaned under a microscope, then subjected to chemical analyses. The two chemical analyses (Fourier-transform infrared (FTIR) and Pyrolysis gas chromatography mass spectroscopy (Pyr-GC-MS)) are common techniques used to chemically profile the fossil resin to give an indication of the botanical affinity.

### Solid-state Fourier-transform infrared (FTIR) analysis of amber

Solid-state Fourier-transform infrared analysis was performed on freshly powdered samples of amber from a cone portion included in potassium bromide pellets. A Perkin Elmer 1600 Series FTIR Spectrophotometer was used in the wavelength range 2.5–15 μm (4000–670 cm^− 1^).

### Pyrolysis gas chromatography mass spectroscopy (pyr-GC-MS) of amber

Pyr-GC-MS analyses were completed using a CDS 2500 Pyrolyzer coupled to an Agilent 5890/5973 GC-MS. Pyrolysis was carried out at 480 °C for 10 s in the presence of excess tetramethyl ammonium hydroxide (TMAH). Pyrolysis products were separated using a 60 m ZB-1701 capillary GC column using He as carrier gas in constant flow (1 ml/min) mode and temperature programmed as follows: 40 °C (4 min), 4 °C/min, 280 °C (16 min). Individual analytes were identified by comparison of MS and retention time data with data for known analytes.

### Pollen sampling and preparation

Samples of the dark remains of the cone fragment were removed using a sharpened needle and prepared for both scanning electron and light microscopy. For scanning electron microscopy (SEM), small pieces of the tissue were transferred to a carbon-covered SEM mount using a wet hair from a superfine brush. The stubs were sputtered with platinum–palladium (2 × 120 s at 20 mA, 10 nm coat thickness) using an automatic sputter coater (Canemco Inc.) and examined under a field emission scanning electron microscope (Carl Zeiss LEO 1530 Gemini).

For light microscopy (LM), the samples picked with needles were treated with Schultze solution (concentrated nitric acid saturated with a few potassium chlorate crystals added) and washed in distilled water, then treated with 4% ammonia and washed until neutral. The extracted material was then mounted on microscopy slides in glycerine jelly, and covered with a coverslip. These pollen preparations were observed with a transmitted light microscope (Carl Zeiss Axioscope A1) equipped with a Canon 450D digital camera, and are deposited with the source specimen (MB.Pb.1997/1246).

### Individual pollen grain preparation

In order to accurately describe the in situ pollen and its range of micromorphology, individual pollen grain preparation was done. Grains were removed from one LM slide (concentrated HCl was used to dissolve the glycerine jelly mounting medium) and the grains picked off. These selected grains were acetolysed (9 parts acetic anhydride, 1 part concentrated sulfuric acid, heated to 100 °C for 4 mins) in a drop on a glass slide, then washed and transferred to a glycerine drop for mounting using a dissecting needle with a nasal hair affixed [38, 39]. After LM photography, the individual grains were mounted on an SEM stub with a drop of absolute ethanol to remove all traces of glycerine. The stub was sputtered with gold. These grains were then observed using a FEI Inspect S50 SEM and then remounted in a different orientation, resputtered with gold and observed again.

## Results

We describe a new fossil-genus and species, based on pollen cones with both in situ amber and pollen present, from the Crato Formation, and infer their affinity as within the extinct order Erdtmanithecales. We also show that the amber can be classified as belonging to the most common type in the geosphere using pyr-GC-MS, highlighting that there were more source plants for this amber type than previously recognized.

### Systematic paleontology

SPERMATOPHYTA.

ERDTMANITHECALES E.M.Friis et K.M.Pedersen, 1996.

Family unknown.

### Remarks

The order Erdtmanithecales was created to accommodate the family Erdtmanithecaceae E.M.Friis et K.R.Pedersen 1996. This family is based on isolated reproductive organs including dispersed pollen of the *Eucommiidites*-type, microsporangiate organs with in situ *Eucommiidites*-type pollen and chlamydospermous seeds with *Eucommiidites*-type pollen in the micropyle of some seed specimens. This newly described microsporangiate taxon has in situ *Eucommiidites*-type pollen, placing it within the order Erdtmanithecales. However, the new taxon described here does not fit well into the family Erdtmanithecaceae since this has been diagnosed with the ‘male reproductive organs of closely spaced microsporangiate units; microsporangiate units stalked and peltate, bearing numerous sporangia in a radial arrangement around stalk; dehiscence of sporangia by longitudinal slits’ [40]. The new taxon has quite a different arrangement. We place the new taxon within the order, but choose neither to amend the Erdtmanithecaceae nor to create a separate family at this time. We highlight the enigmatic morphology of the cones being markedly distinct from all previously recognized microsporangiate organs producing *Eucommiidites*-type pollen. Much more diversity among Erdtmanithecales is also inferred from the diversity of conspicuously differently sized isolated seeds known from various Early Cretaceous sites [10].

*Araripestrobus* Seyfullah, E.A.Roberts, A.R.Schmidt et L.Kunzmann gen. nov.

*Etymology*: From the Araripe Basin, where the Crato Formation flora originates.

*Generic diagnosis*: Large (up to 170 mm long and 74 mm wide) microsporangiate pedunculate cones, appearing ‘segmented’ or organized into distinct layers in lateral outer surface view, built up of about 10 disc-shaped cone portions and a basal and apical cone portion with dome-shaped apex and base respectively, gaps present between the layered portions. Peduncle robust, short, diameter about 1/3 of the cone diameter. Detached microsporophylls leave rhomboidal scars with a central impression of a possible vascular bundle. Individual cone portions found detached more or less trapezoid to diamond-shaped in cross-section with a longer and a shorter diameter and the cone axis sub-centrally; outer margins frequently arc-shaped in cross-sectional view. Slender, wedge-shaped microsporophylls, in dense facetted arrangement, generally in helical phyllotaxis; particularly each cone portion with 2–4 levels of microsporophylls, in alternating positions in adjacent levels. Each microsporophyll with 10 or more longitudinally running resin canals. In situ pollen of *Eucommiidites*-type, boat-shaped, ‘trisulcate’, surface finely perforate, smooth to microrugulate; microfossulae between perforations.

*Type*: *Araripestrobus resinosus* gen. nov. et sp. nov.

*Araripestrobus resinosus* Seyfullah, E.A.Roberts, A.R.Schmidt et L.Kunzmann gen. nov. et sp. nov.

*Etymology*: The specific epithet highlights the resinous nature of the cone.

*Holotype*: specimen MB.Pb.1997/1246, illustrated in Fig. 3a.

*Paratype 1*: specimen SM. B 16594, illustrated in Fig. 2a.

*Paratype 2*: specimen MB.Pb.1997/1350, illustrated in Fig. 2b-e.

*Type horizon and locality:* Lower Cretaceous (upper Aptian-lowermost Albian), C6 limestone horizon, Crato Formation, Santana Group, Araripe Basin, northeast Brazil.

*Specific diagnosis*: as for genus.

**Fig. 1:**
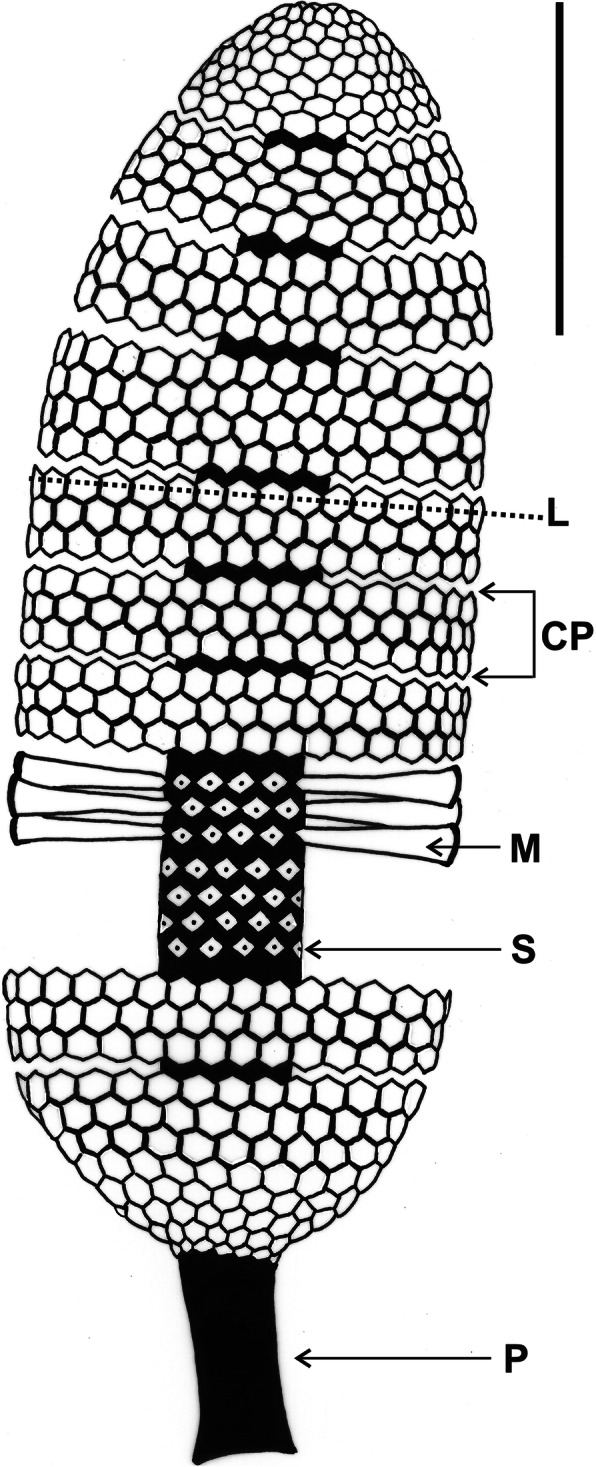
Reconstruction of *Araripestrobus resinosus* gen. nov et sp. nov. with terminology used here: L – level of microsporophylls (horizontally aligned microsporophylls, not congruent with phyllotaxis); CP – cone portion (if isolated – detached cone portion); M – microsporophyll; S – scar of detached microsporophyll on cone axis (dot – putative vascular bundle); P – peduncle; scale bar: 50 mm

Remark to the selection of types: The most significant specimen displaying almost all diagnostic characters is selected as the holotype. But, it is only a disarticulated cone fragment, herein called a detached cone portion (for terminology used see Fig. 1), lacking important gross-morphological features. Therefore, an almost complete cone is additionally selected as the paratype 1 and an incomplete cone, a key specimen illustrating the initial disintegration of cones into distinct portions as well as resin canals of microsporophylls is selected as paratype 2.

*Additional material*: Detached cone portions: FPH-83-B; MB.Pb.1999/1440; SM.B 16599; UOP-PAL-MC0004. Complete cone: MB.Pb. 2020/0020 (rubber cast).

#### Description

**Fig. 2:**
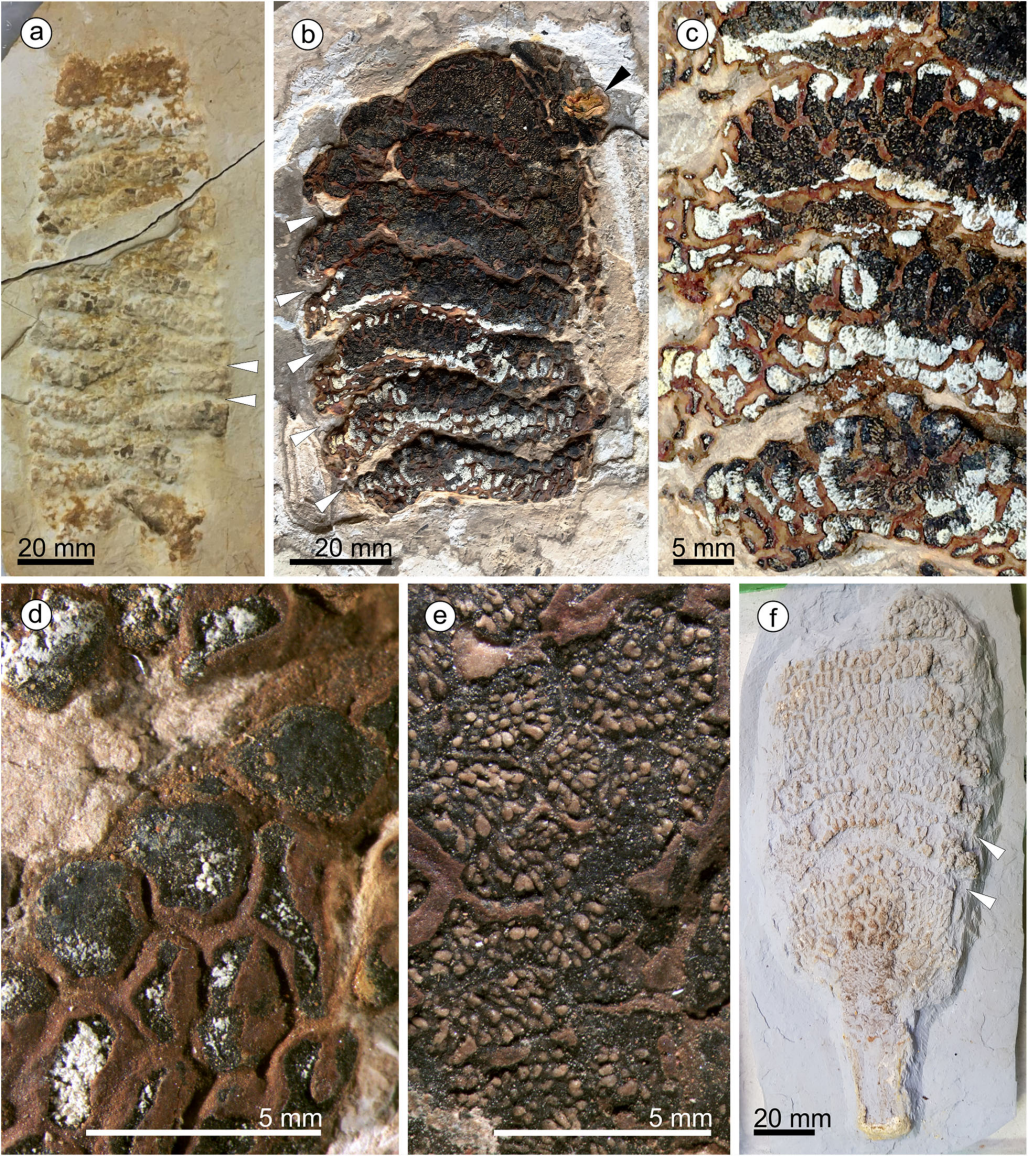
Longitudinally preserved cones of *Araripestrobus resinosus* gen. nov et sp. nov. from the Crato Formation**. a** Paratype 1 (SM.B 16495), almost complete cone in lateral outer-surface-view, showing sigmoidal-shaped gaps between groups of layered microsporophylls in the lower part of the cone (arrowheads). **b**–**e** Paratype 2 (MB.Pb.1997/1350). **b** A median-apical cone part with seven cone portions with a gap between them (white arrowheads), and a rounded dome-shaped apex with a bleb of amber towards the right side of the specimen apex (black arrowhead). **c** Median cone part displaying individual cone portions with two to four horizontal rows of microsporophylls and a gap between these portions. **d** Polygonal-hexagonal outline of cross-sections of microsporophylls. **e** Upper cone part showing microsporophylls with exposed resin canals. **f** A rubber cast (MB.PB.2020/0020) of a basal-median part of a cone including a peduncle, arrowheads indicate gaps developing between microsporophyll layers

**Fig. 3:**
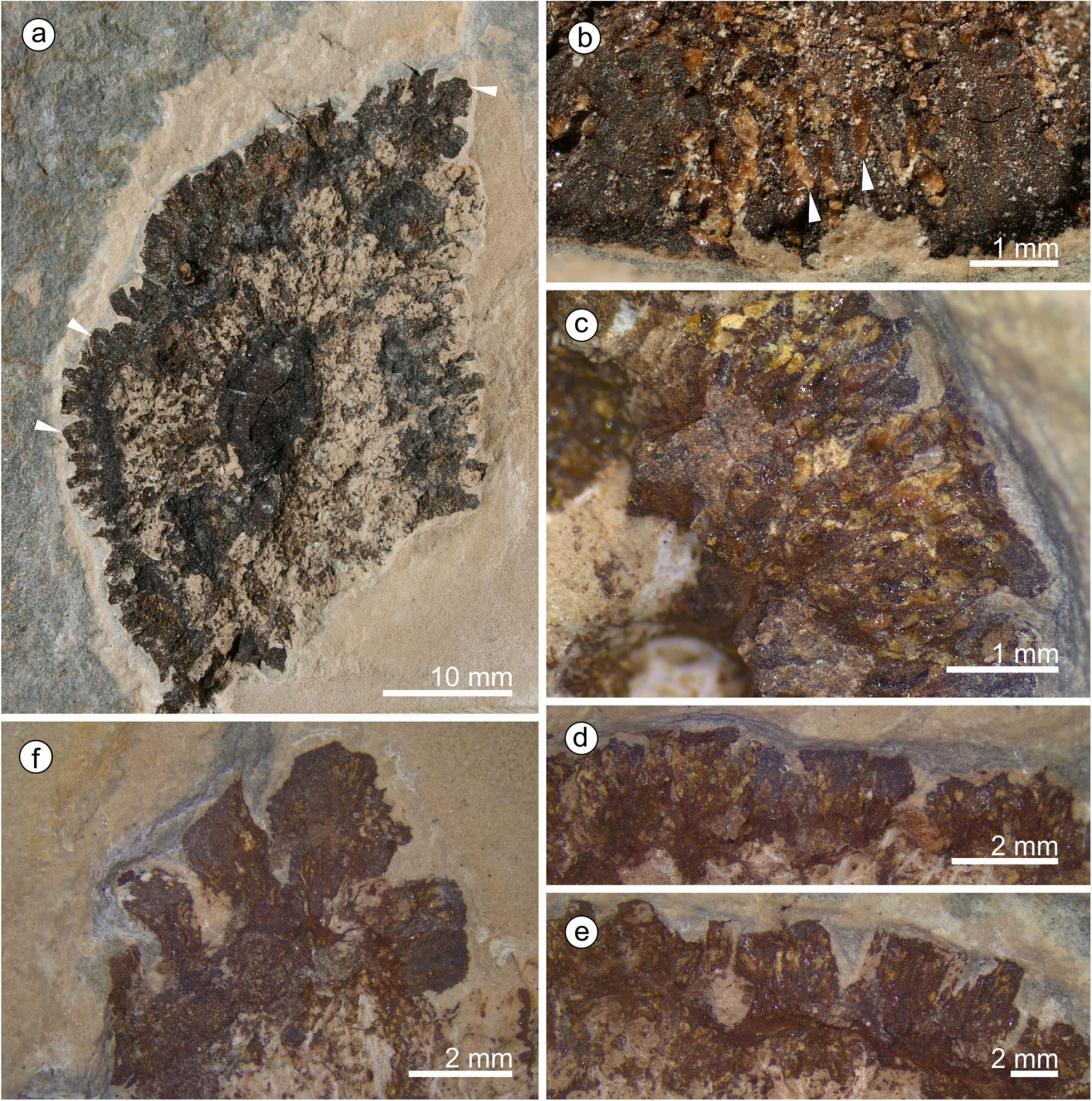
Holotype of *Araripestrobus resinosus* gen. nov et sp. nov. (MB.Pb.1997/1246) from the Crato Formation with in situ pollen and amber present. **a** Apically compressed detached cone portion with central axis (dark central oval) and the remains of microsporophylls (arrowheads). **b** Variously coloured amber pieces embedded within the darker brown plant tissue remains at the margin of the portion, amber clearly in rows (arrowheads) indicating the position of resin canals. **c** Resin canals infilled with fossil resin within the plant tissue remains showing that the densely packed canals run from the microsporophyll margin towards their interior (3 stacked images). **d**–**f** Microsporophylls with amber and various outlines (**e** 9 stacked images, **f** 5 stacked images)

**Fig. 4:**
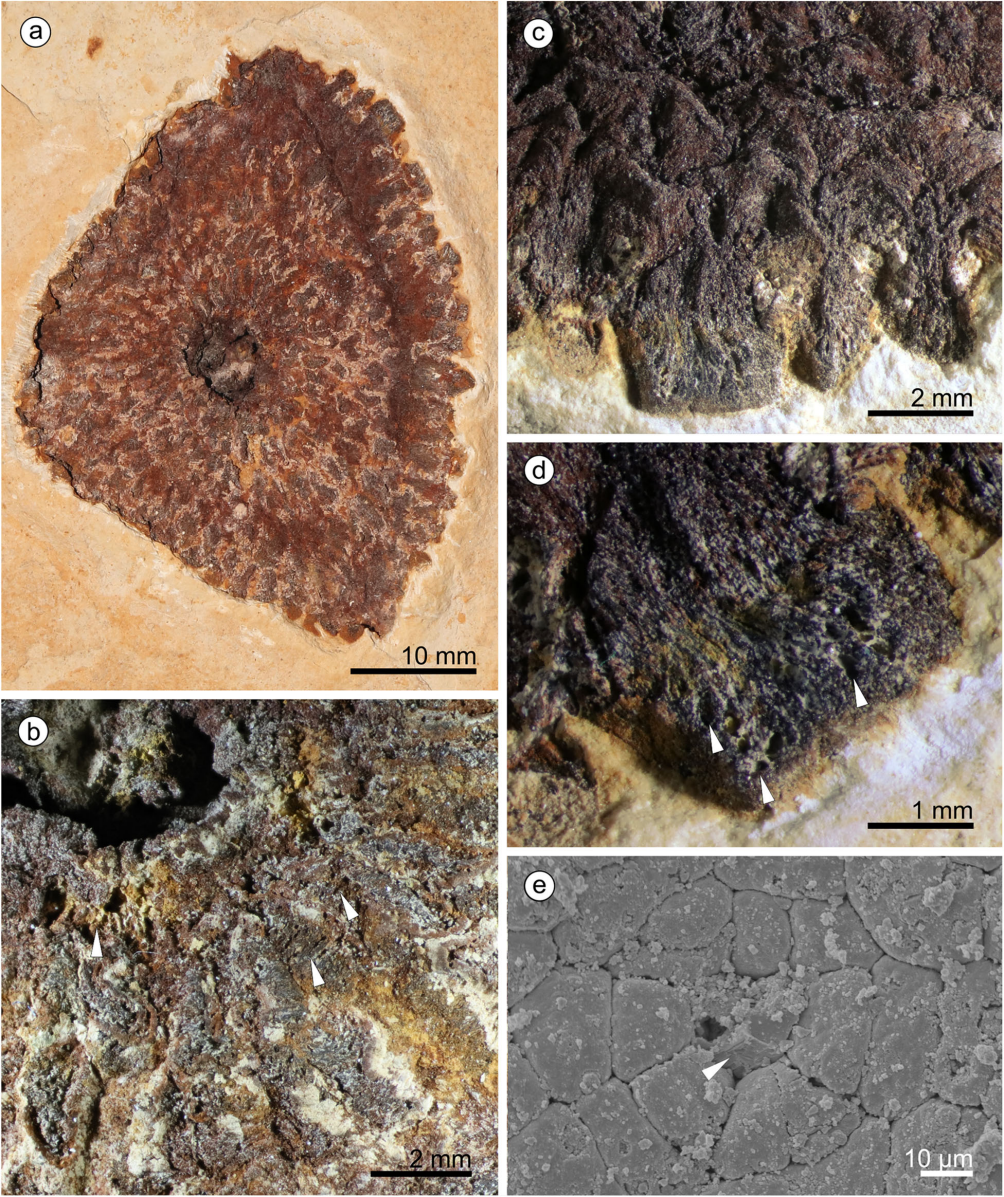
*Araripestrobus resinosus* gen. nov et sp. nov. from weathered material (UOP-PAL-MC0004) **a** Apically compressed detached cone portion with central axis (dark central oval) and the remains of microsporophylls. **b** Microsporophyll remains radiating from the central axis (upper left side), arrowheads indicating fibrous nature of microsporophylls. **c** Better preserved microsporophylls at the edge of the detached cone portion showing more detail of the microsporophyll shape and structure. **d** Detail of a thick microsporophyll with now empty resin canals seen in oblique view as oval voids (arrowheads). **e** Scanning electron micrograph of a microsporophyll showing resin (arrowhead) inside a canal

**Fig. 5:**
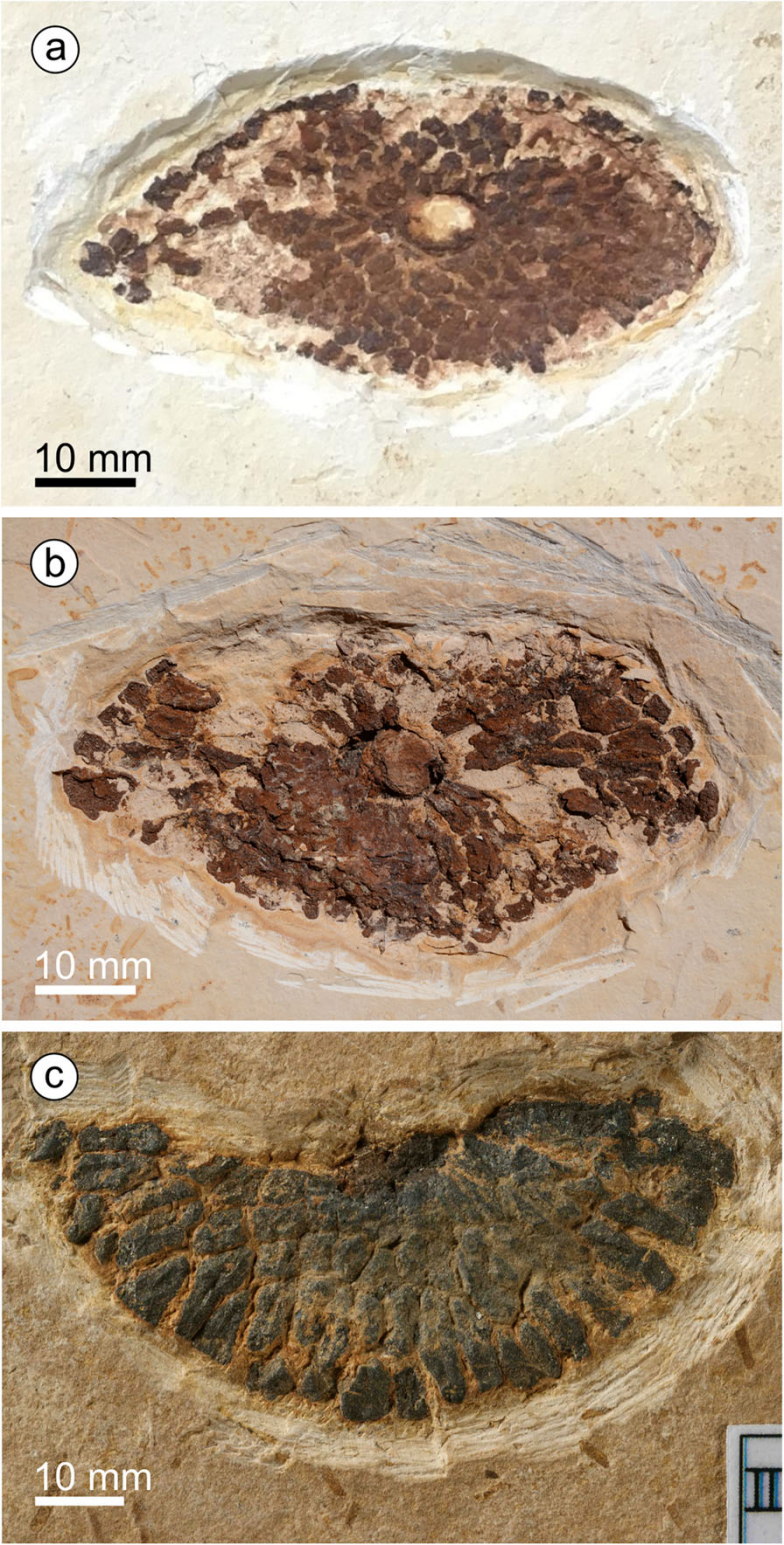
Other detached portions of cones assigned to *Araripestrobus resinosus* gen. nov et sp. nov. **a** Specimen SM.B. 16599 is an almost completely preserved rather diamond-shaped detached cone portion. **b** Specimen FPH-83-B is an ovoid-diamond-shaped detached cone portion in cross-sectional view. **c** A fragment of a detached cone portion, MB.Pb.1999/1440

**Fig. 6:**
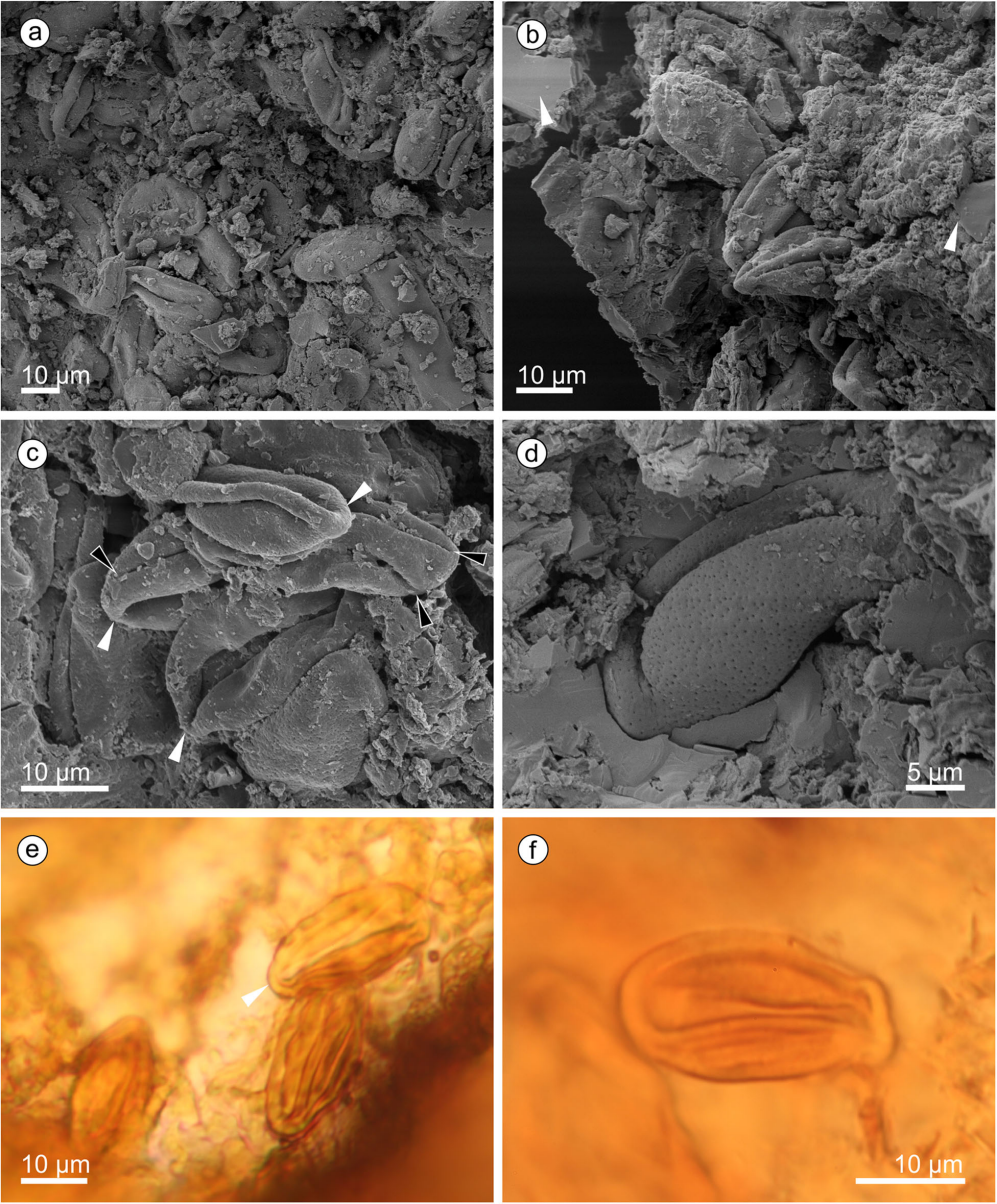
*Araripestrobus resinosus* gen. nov et sp. nov in situ pollen. **a**–**d** Scanning electron micrographs, **e**–**f** light micrographs. **a**–**b** Pollen grains embedded in the cone tissue, arrowheads indicate areas of preserved amber. **c** Pollen grains in different orientations showing typical main sulcus with rounded ends (white arrowheads) and lateral pseudosulci with sharp ends (black arrowheads). **d** Amber-embedded pollen grain showing a lateral sulcus and the finely scabrate, punctate ornamentation; **e**–**f** Pollen grains adhered to microsporangial surface showing the sulci with rounded ends (arrowhead)

**Fig. 7:**
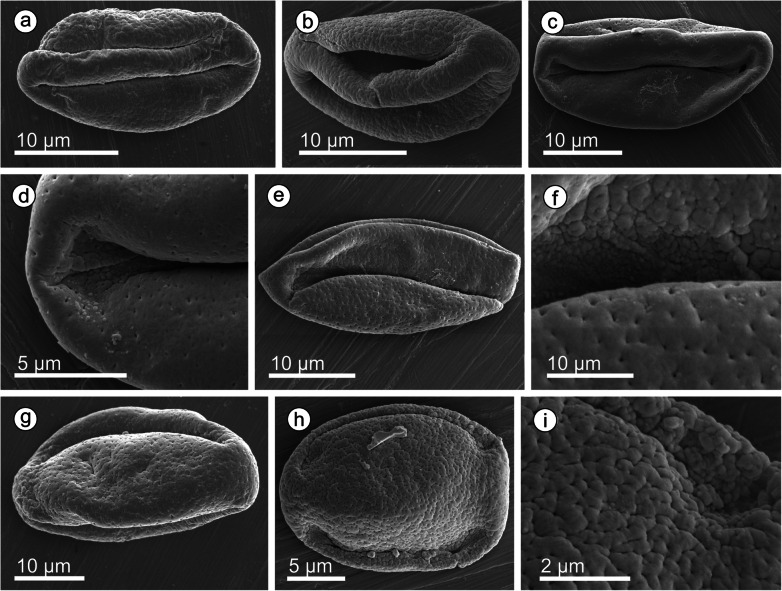
Individually prepared pollen grains from *Araripestrobus resinosus* gen. nov et sp. nov. under SEM. **a**–**c** Pollen with view of the sulcus. **d** Detail of c showing smooth perforate sulcus membrane and rounded end. **e** Pollen grain folded along sulcus showing one pseudosulcus and the perforate microrugulate surface. **f** Detail of pseudosulcus area near rounder end from grain in e. **g**–**h** Pollen orientated on the sulcus with the two pseudosulci visible and variable surface texture. **i** Detail of h showing the microrugulate surface with both microfossulae and perforations

We assign eight specimens to the new genus and species, and provide a reconstruction to aid understanding of our terminology and interpretation due to the unusual morphology of the cones and microsporophylls (Fig. 1). There are three almost complete cones (Fig. 2) and five disc-like structures that we term ‘detached cone portions’, which are fragmentary remains of cones where a portion from the cone has become detached, is now observed in transverse section, and is composed of several layers of microsporophylls that are usually preserved around a cone axis. One detached cone portion (holotype, Fig. 3a) is from unweathered material and so has both amber and pollen present in situ. The four other detached cone portions share a similar gross morphology and they are preserved in the same orientation with the central axis perpendicular to the bedding surface, giving a compressed transverse view of the detached cone portions. All the detached cone portions are at least partially three-dimensionally preserved, with varying amounts of tissue remaining (Figs. 4 and 5). Due to the partial nature of these cone remains it is not possible to determine the original shape and any abaxial or adaxial surfaces in these specimens. Only the holotype has yielded in situ pollen (Figs. 6 and 7). The almost complete cones, one of which is the paratype 1, represent flattened organs in longitudinal view. Paratype 1 is almost an impression with nearly no coalified tissue remaining (Fig. 2a); the second cone specimen, paratype 2 (Fig. 2b-e), preserved as goethite replacement, nicely exhibits the outer cone surface and the beginning of disarticulation into the characteristic portions (Fig. 2b arrowheads mark areas of separation between the portions); and the third specimen is a rubber cast from a cone from which microsporophylls are partly detached displaying the cone axis and the phyllotaxis of microsporophylls (Fig. 2f). Based on the identical microsporophyll morphology of the detached cone portions and the almost complete cones, all fossils belong to the same taxon.

#### Complete cones

These three laterally compressed specimens are more or less cylindrical in outline, but none are complete. Given the unusual morphology of the cones we provide a diagrammatic reconstruction (Fig. 1) based on a composite of these specimens to aid with terminology and interpretation. Paratype 1 (SM.B 16495, Fig. 2a) is an almost complete cone in lateral outer-surface-view 170 mm long and up to 66 mm wide. Its shape is cylindrical to slightly ovoid with widest width after approximately one-third of the cone length. The apex and base are not completely preserved but from a geometrical point of view, and compared to the other specimens, only small parts are missing. It is composed of 12 different layers of grouped microsporophylls that we equate with the smaller dispersed cone portions. The horizontal outline of some of the median portions is somewhat sigmoidal but the outline of the apical portions is rather horizontal in relation to the long cone axis (Fig. 2a). Individual portions are 10–12 mm high, only the uppermost portion measures 16 mm in height and is not complete. Each portion contains at least two adjacent levels of microsporophylls with their shapes corresponding to the shape of those from isolated cone portions described above. We call a horizontal set of microsporophylls a ‘level’ (Fig. 1) instead of a ‘row’ because the latter term could be misleading implying a whorled phyllotaxis of microsporophylls. Individual portions of the cone are markedly separated by a narrow gap, giving the segmented or layered appearance (Fig. 2a). The overall shape and arrangement of microsporophylls at the cone appears facetted. The outline of the distal ends (heads) of microsporophylls is almost hexagonal isodiametric but appears somewhat irregular most likely due to taphonomic deformation of the cones, the surface is almost smooth. The phyllotaxis of the microsporophylls within a cone segment is not clearly observable; however, the alternating arrangement of microsporophylls of adjacent levels excludes arrangement in vertical rows (orthostichies).

The apical part of the cone is best seen in Paratype 2 (MB.Pb.1997/1350, Fig. 2b), measuring 105 mm long and 63 mm wide, with seven cone portions present. The apical cone portion shows a rounded dome-shaped apex and consists of more horizontal microsporophyll levels than the other portions, about 5–6. While the apical cone portion is 19 mm high, the remaining portions are 10.5–15 mm high. The median cone part shows distinct wide gaps at the cone margin, that narrow towards the cone axis, between the individual cone portions. Medial cone portions are three to four microsporophyll levels thick (Fig. 2c). The median cone portion also displays the facetted arrangement of microsporophylls, which are polygonal-hexagonal in cross-section (Fig. 2d). Microsporophylls of the upper cone portions show numerous resin canals per microsporophyll; the arrangement of which is likely in a circle around the microsporophyll’s center (Fig. 2e). A bleb of amber is seen towards the right side of the specimen apex in contact with a cone portion fragment which is obviously disarticulated from this or from another cone. Therefore, it is not clear whether the amber originated from this specimen.

The basal part of a cone including a peduncle is best observed in the third specimen, a rubber cast (MB.PB. 2020/0020, Fig. 2f), measuring 130 mm in length and 74 mm in width. The peduncle part is preserved displaying no features of previously attached leaves. The massive peduncle is about 20 mm in diameter but could be somewhat flattened and about 42 mm long. Eight cone portions are preserved, the basal most being somewhat higher than the remaining (10.5–15 mm). Individual cone portions display 2–4 levels of microsporophylls, in alternating arrangements between adjacent levels. Considering the cone dimensions, the microsporophylls are approximately 15–20 mm long. Some microsporophylls from the basalmost cone portions are detached showing the “naked” cone axis with densely arranged rhomboid scars in helical phyllotaxis. The flattened preservation of the cone does not allow us to determine parastichy numbers.

#### Detached cone portions

The detached cone portions show cross-sectional internal cone morphology. Their general outline varies from ovoid-diamond-shaped (Figs. 3a, 5b), to roughly triangular (Fig. 4a), to almost oval (Fig. 5a). The outer margins appear arc-shaped (Fig. 5a, c) and roof-shaped (Figs. 3a, 4a); both shapes can be present in one specimen (e.g. holotype, MB.Pb.1997/1246; Fig. 3a). Additionally, the holotype exhibits a small indentation on one side (Fig. 3a). Length and width of the detached portions are always dissimilar (see Table 1) measuring 45–65 mm by 21–42 mm. The central to sub-central axis is rounded to oval in cross-sectional outline (for measurements see Table 1). The central axis appears to be slightly sunken from the plane of the microsporophylls and slightly contracted away from them.

**Table 1:** Dimensions and preservation state of detached cone portions of *Araripestrobus resinosus* gen. nov. et sp. nov.

Character Specimen (figure reference)	Preservation	Outline / shape of cone portion	Length [mm]	Width [mm]	Size of axis in cross-section [mm]
**MB.Pb.1997/1246** (holotype; Fig. 3a)	compression: cuticle, in situ pollen, in situ resin	ovoid-diamond-shaped	57.4	34.6	7.5 × 13.2
**UOP-PAL-MC0004** (Fig. 4)	oxidized no in situ resin	almost triangular	54	42	6 × 12
**SM.B. 16,599** (Fig. 5a)	oxidized no in situ resin	almost diamond-shaped	61	29.5	6.5 × 6.5
**FPH-83-B** (Fig. 5b)	oxidized no in situ resin	ovoid-diamond-shaped	65	32.5	8 × 8
**MB.Pb.1999/1440** (Fig. 5c)	compression: in situ resin, no cuticle, no in situ pollen	half portion: arc-shaped	45.5	21	(not preserved)

Tightly packed microsporophylls radiate out from the axis forming up to four overlapping levels. The distal level slightly slopes up to the sediment surface. Phyllotaxis cannot be deduced from the detached cone portions; at least the microsporophylls of adjacent levels seem to be in alternating positions. Microsporophylls are slender wedge-shaped; the proximal part is not widened like an apophysis but the outer margin is very slightly curved. If the outer surface is somewhat abraded the margin appears irregular and rugged/fissured. These distal parts are up to 4.5 mm wide. The likely most complete microsporophyll is 13.5 mm long in longitudinal lateral view. Each microsporophyll contains several longitudinally running resin canals (average 0.1–0.2 mm wide in longitudinal section) filled with a yellow-orange-to red-brown resin. Resin ducts situated within the original plant tissues (in situ amber) are apparent in the holotype (Figs. 3b-e) and in specimen MB.Pb.1999/1440. The number of resin canals is difficult to count but at least more than 10 canals per microsporophyll are present. The ovals of empty channels, seen in specimen UOP-PAL-MC0004 (Fig. 4d), measure 0.1–0.2 mm in width and 0.1–0.3 mm length.

#### In situ pollen grains

Despite pollen sacs not being recovered, in situ pollen grains (Fig. 6) were found clustered within the plant tissue (Fig. 6a), alongside pieces of in situ amber (Fig. 6b, arrowheads). The pollen is ovoid to elliptic in outline in equatorial view (22–35 μm long, 10–20.5 μm wide), with a broad sulcus that is often almost closed over by the rest of the surface and has rounded ends (Fig. 6c white arrowheads). In some orientations, the pollen grains are quite folded and even almost appear to have further furrows (Fig. 6c, black arrowheads), but these are not always present. Under SEM, the pollen often has a perforate surface becoming finer to almost smooth towards the sulcus (Fig. 6c-d). Extracted pollen grains are found adhered to the remains of the microsporangial walls (Fig. 6e-f) and are clearly boat-shaped and variously folded. Individually extracted grains (Fig. 7) show a range of lengths and surfaces ornamentation. The grains are 19–30 μm in maximum length (using SEM, x = 4). They are boat-shaped and ‘trisulcate’, i.e. monosulcate with two pseudosulci present. The sulcus has a membrane that can be microrugulate to smooth and finely perforate (Fig. 7a-d). The remaining surface is finely perforate and can be smooth to almost microrugulate (Fig. 7e-g) between the perforations; sometimes there are microfossulae (Fig. 7h-i). Based on the combination of these features, the pollen closely resembles the dispersed *Eucommiidites*-type pollen.

### Fourier-transform infrared (FTIR) analysis of Crato formation ambers

**Fig. 8:**
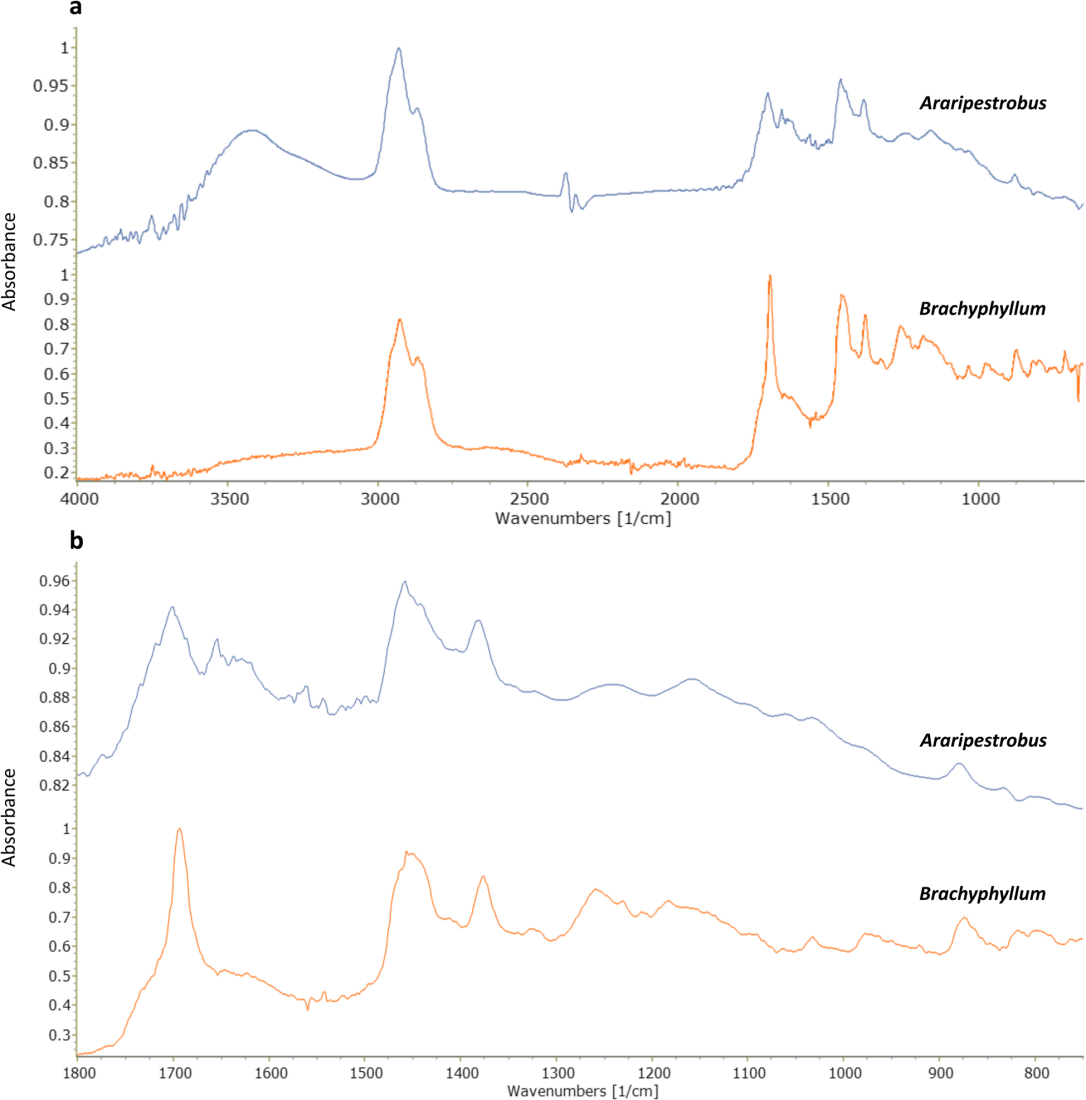
FTIR spectra of extracted in situ Crato ambers from *Araripestrobus resinosus* gen. nov et sp. nov. and *Brachyphyllum obesum*. **a** Full FTIR spectra. **b** Detail of the fingerprint region of the spectra

The FTIR spectra of both the extracted in situ amber from *Araripestrobus* gen. nov. and of the *Brachyphyllum* specimen (Fig. 8) are typical for fossil resins. The important insight is that the *Araripestrobus* gen. nov. spectrum is quite distinct from that of *Brachyphyllum*. The main features of the spectra are reported in Table 2.

**Table 2:** Main absorption peaks (FTIR spectra) of extracted ambers from plant fossils from the Crato Formation

Functional group	Band, wavelength [μm]	Band, wavenumber [cm^−1^]	Intensity	Assignment
O-H	2.95	3390	medium	Stretching of O-H bonds
-CH_2_ -CH_3_	3.40	2940	strong	Stretching of C-H bonds
-CH_2_ -CH_3_	6.90	1450	medium	Scissoring and bending of C-H bonds
-CH_2_ -CH_3_	7.25	1380	medium	Bending of C-H bonds
-CH_2_ -CH_3_	11.39	878	weak	C=CH_2_: out-of-plane bending of H in terminal methylene group
-C=O	5.80	1724	medium	Stretching of C=O double bonds
-C-O-	8.15, 8.60	1227, 1163	medium/weak	Absorption of C-O single bonds
-C-O-	9.60, 9.85	1042, 1015	weak	Absorption of C-O single bonds (alcohol)
C=C	6.05	1654	weak	C=C stretching
-C-H aromatic	12.10, 14.10	826, 709	weak	Out-of-plane bending of aromatic C-H

The broad absorption band with a maximum near 3400 cm^− 1^ in *Araripestrobus* gen. nov. is due to stretching motions of O-H bonds, in part explained by atmospheric moisture absorbed by the sample during the preparation for the analytical procedure, or as result of oxidation by atmospheric oxygen [41]. In both spectra, the strong band at 2940 cm^− 1^ is due to the stretching of aliphatic carbon-hydrogen bonds [2] and is considered diagnostic of resinous structures [42]. Bending of the same bonds produces the absorption peaks at around 1450 cm^− 1^ and 1380 cm^− 1^ [2]. Another absorption band is at around 1700 cm^− 1^, the so-called “carbonyl band” [2], also a feature typical of all fossil resins, caused by stretching of carbon-oxygen double bonds.

The above described absorption bands are found in all fossil resins, and therefore are not of particular diagnostic interest, but confirm the occurrence of a fossil resin. The upper part of the infrared spectrum, higher than 1250 cm^− 1^, is difficult to interpret in terms of chemical structure [2], but it is more useful than the lower region of the spectrum because it varies among different resins. In the FTIR spectra of the amber, the region between 1250 and 1000 cm^− 1^ shows absorption bands caused by carbon-oxygen single bonds [2, 43]. The vibrations of these bonds are influenced by the carbon skeleton of the whole molecule, and so it is difficult to assign the absorption bands of this region to a specific structure. However, the region from 1250 to 625 cm^− 1^ is particularly useful as a “fingerprint” of any amber [2].

An additional weak band at (1654 cm^− 1^), due to stretching of double carbon-carbon bonds was found in *Araripestrobus* gen. nov. but appears as a shoulder on the peak at 1690 cm^− 1^in the *Brachyphyllum* amber. Another very weak absorption band was found at 878 cm^− 1^ in *Araripestrobus* gen. nov. and in *Brachyphyllum* ambers. This band, compatible with the 11.3 μm band described by [2, 44], is due to out-of-plane bending movements of two hydrogen atoms in a terminal methylene group, as a feature of resin acids. This band is typically lacking or weak in older resins, but can also be due to rapid oxidation of the methylene group after exposure to atmospheric oxygen [2, 44].

### Pyrolysis gas chromatography mass spectroscopy of in situ amber from *Araripestrobus resinosus* gen. nov. et sp. nov

**Fig. 9:**
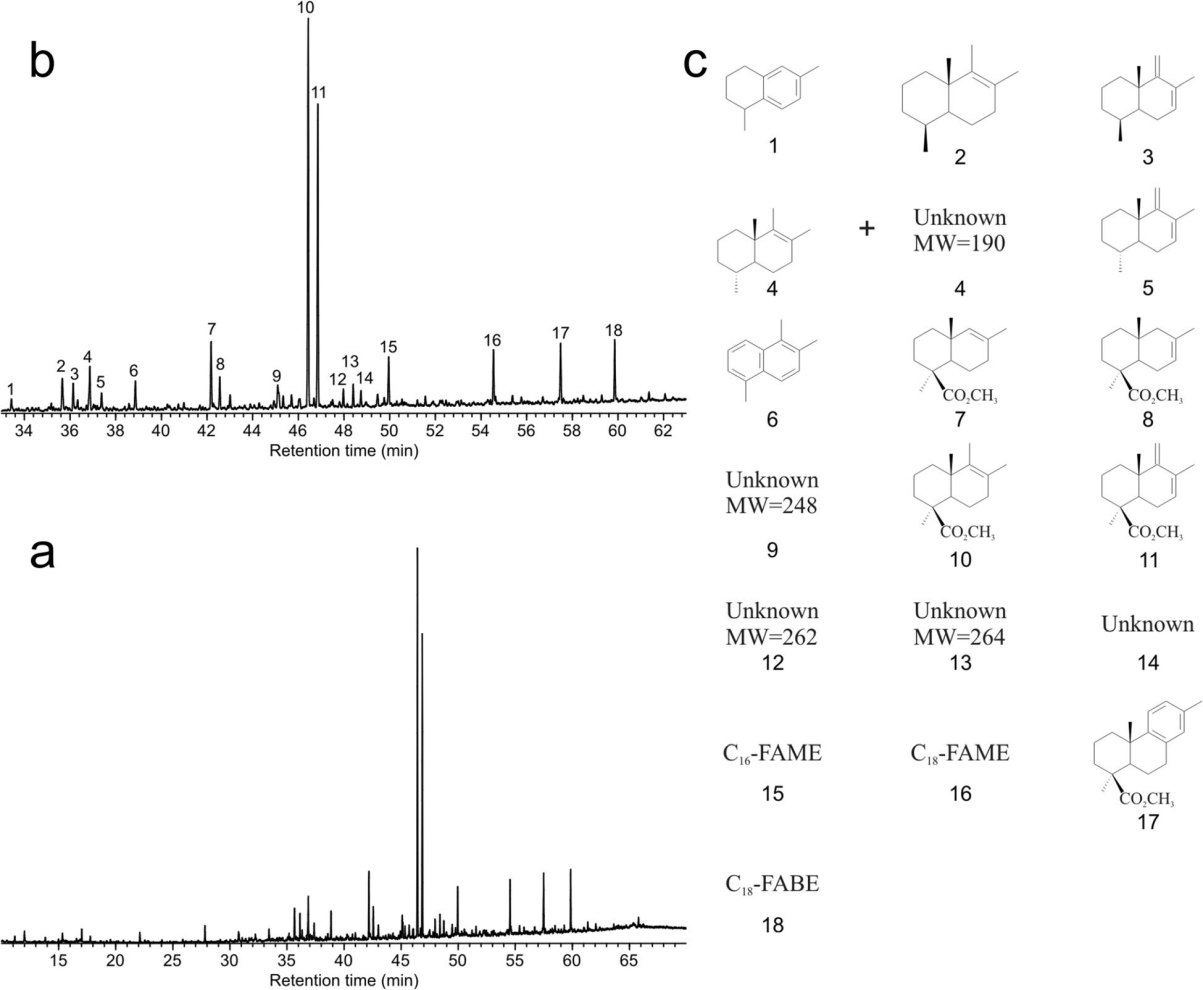
Ion chromatograms of in situ *Araripestrobus resinosus* gen. nov et sp. nov. amber analyses with Pyr-GC-MS. **a** Total ion chromatogram from the sample. **b** Detail of the chromatogram with numbered peaks for identification. **c** Structures identified, numbers correspond to those numbered peaks in b

The amber is a mature class Ib amber, which has a regular polylabdanoid structure lacking succinic acid, with minor amounts of various fatty acid products also present, but no palaeobotanically-useful biomarkers (Fig. 9).

## Discussion

### Palaeobotanical significance of *Araripestrobus* gen. nov

#### Cone architecture

Based on the conspicuous morphology of the slender wedge-shaped microsporophylls and their facetted arrangement (see Figs. 1, 2a, 3c, 4), both the almost complete cone specimens (Fig. 2) and the detached cone portions (Figs. 3, 4 and 5) belong to the same taxon without any doubt. The long axes of the detached cone portions (54–65 mm) correspond in their dimensions to the widths of the cones (maximum width 63–74 mm). From the complete cones it is visible that each cone portion contains two to four horizontal levels of microsporophylls (see Fig. 1 for terminology); the same number of levels is observed from the detached cone portions which display the cone in cross-sections.

The different shapes of the basalmost and apicalmost cone portions could putatively depend on a denser arrangement of not fully developed cone portions, but the complete cones are not well enough preserved for a confident statement of this fact. Our conclusion, that cones with their layered appearance of groups of microsporophylls and we find these layered parts as disc-shaped remains when preserved as transverse detached cone portions, implies that either these disc-shaped portions are easily shed from the cone axis, or the cone including its axis disintegrated into these portions. The first hypothesis is supported by the observations that (1) the paratype 2 displays the state of beginning disarticulation of cone portions and (2) that some cone portions lack axis tissue but show a hole instead. Although the cone peduncle superficially appears to be robust and the cone itself too, simply based on its size, the original tissue was obviously much softer than compared to fully woody organs. The sigmoidal deformation of the cone portions, as seen in the complete cones (Fig. 2), and the lateral-longitudinal flattening of the complete cones indicate a non-woody or not completely woody nature of these microsporangiate organs. A single specimen (Fig. 2f) displays parts of the “naked” cone axis. It clearly indicates a helical phyllotaxis of the microsporophylls but does not expose whether the cone axis is segmented or not, corresponding to the layered groups of microsporophylls anticipating disintegration into cone portions. Calculated from the diagrammatic reconstruction of a complete cone based on a composite of specimens, a detached cone portions encompasses about 120–160 microsporophylls, and a complete cone about 1450–1850.

Furthermore, we are convinced that these cones are microsporangiate organs because they lack any ovule or seed but yield clusters of pollen. The detailed biology of these organs cannot be completely described because the original location where pollen was produced, the pollen sacs (microsporangia), cannot be clarified with the studied specimens. If the detached cone portions are indeed shed from the axis they were obviously mature and pollen was likely at least partly already released. That could be the reason why not all detached cone portions yield in situ pollen in addition to being found in weathered sediments. Another possibility is that as the mature cone degrades the microsporophylls start to ‘clump together’ building distinct horizontal gaps and form these segmented portions as the cone decays. Gaps could be formed to better release pollen which could also explain why there are no pollen sacs left in the detached cone portions. Invisibility of (free) pollen sacs in our cone material could be a hint that pollen sacs were embedded in microsporophyll tissue, similar to elongated embedded pollen sacs in *Erdtmanitheca portulacensis* [45]. Unfortunately, internal tissue of the microsporophylls is not preserved in our studied detached cone portions.

Based on cone architecture, with the cone appearing to have layered groups of microsporophylls, and disintegrating into distinctive detached portions of non-peltate microsporophylls, the new taxon does not resemble any known fossil taxon, neither described from the Crato Formation flora nor from other Cretaceous assemblages. Its characters are also clearly dissimilar from any pollen-producing structure in extant gymnosperms, of which Cycadales, Gnetales and Pinales are briefly discussed below. In particular, the cone architecture is rather dissimilar to any known cone types from late Mesozoic conifers and gnetaleans as well as from microsporangiate organs of early angiosperms. Only bennettitalean and cycadalean structures seem to have a superficially similar architecture but are bisexual structure in case of bennettitaleans. These pollen cones give evidence of a hitherto unknown gymnospermous plant within the Crato Formation, however, the affinities of the cones from solely their morphology was unclear. Previous studies of the Crato Formation flora had shown the presence of gnetaleans and conifers [46], and cycadophytes [47], the latter group is only known from the pollen record.

Although the reproductive biology of the microsporophylls present in *Araripestrobus* gen. nov. is not completely known, these simple reproductive units are dissimilar to equivalent organs of extant Gnetales. In *Ephedra*, pollen cones are composed of bracts that enclose stalked antherophores, each of which consists of two fused microsporophylls bearing fused microsporangia [48]. The compound pollen cones of *Gnetum* exhibit a whorled phyllotaxis with collar-like structures with numerous microsporophylls at nodes markedly separated by internodes [48]. Cones of some species bear also non-functional ovules. The compound pollen cone of *Welwitschia* is, by origin, a bisexual organ with the staminate “flowers” and non-functional ovules. Its gross morphology is characterized by dense decussate arrangement of the staminate “flowers” consisting of fused bracts and whorled microsporangiophores [48].

Many gnetalean and gnetalean-like fossils have been described from the Crato Formation flora, those described with reproductive structures have opposite-decussate scaled small, slender pollen strobili, e.g. *Welwitschiostrobus murili* [31]. The two putative gnetaleans from the Crato Formation flora are clearly distinct from our cone. *Cearania heterophylla* also has small slender pollen strobili [22], and *Cariria orbiculiconiformis* has a triplet reproductive structure including a pair of orbicular seed cones and a basket-like pollen strobilus in between the two seed cones [33]. It is clear that a gnetalean affinity for our cones can be ruled out solely based on the very different cone shape and structure, and that no gnetalean has amber preserved in situ. The recovered in situ pollen also has no known gnetalean affinities.

Cycadaleans and bennettitaleans are assumed to be rare or even absent from the megafossil component of the Crato Formation flora. They are proven present from the pollen assemblage [19, 47, 49]. Possible ovulate cones are present, 8 cm long and about 6 cm in diameter, that are possibly referable to the bennettitalean *Williamsonia* [26] but this had not been evidently shown. Other plants with less clear, but still likely gymnospermous affinity have also been described from the Crato Formation flora such as *Novaolindia dubia* [32], which as yet has no microsporangiate organs described. The sole seed fern taxon is thought to have affinity to the Caytoniales as it has multiovulate cupules, but microsporangiate organs are also not known so far [26]. In situ amber has not been reported from any of these plants listed above.

Cone architecture of *Araripestrobus* gen. nov. resembles that of extant cycadalean plants. Rather uniformly, they produce cylindrical cones with numerous apparently vertically but helically arranged wedged-shaped to peltate microsporophylls bearing numerous groups (sori) of small globose microsporangia [50]. At maturity, the cones open in a quite different way in that individual microsporophyll apophyses separate one from another to release pollen through the gaps. Cones are often curved, and finally they are shed as entire entities.

Among the conifers described from the Crato Formation are the Araucariaceae (*Araucaria* and *Brachyphyllum* [9, 27]), and the Cheirolepidiaceae [9, 28, 29], and other taxa of uncertain affinity, such as *Lindleycladus* [27]. From the latter taxon, reproductive structures are still unknown. The *Classopollis* pollen of Cheirolepidiaceae is very distinctive, and pollen cones of this family are comparatively small exhibiting peltate microsporophylls [51]. However, such cones have not been reported from the Crato Formation flora so far. In general, pollen cones of extant Araucariaceae are composed of numerous peltate microsporophylls bearing elongate free microsporangia producing distinctive pollen types while fossil araucarian pollen cones are often rather small with fewer microsporophylls [52]. A putative *Agathis*-like pollen cone with preserved resin canals with in situ fossil resin ([8]; Pl 2, Figs. 1 and 2) and a putative female araucarian cone ([8]; Pl 2, Figs. 3, 4 and 5) were figured from the Crato Formation. What is striking when comparing our pollen cones to that of [8] is that all three have amber infilling the resin canals, but morphologically they are very distinct. *Araripestrobus* gen. nov., despite being preserved in a different orientation, and more usually disintegrates into portions of a larger cone structure, also has differing shaped microsporophylls that are far smaller having hexagonal outlines of the distal ends and smooth outer surfaces. Pinaceae, Podocarpaceae and Cupressaceae are only reported from the pollen record [49]. Male cones of these conifers are far smaller than our cones and they are composed of markedly fewer microsporophylls, which is why any affiliation of *Araripestrobus* gen. nov. to these families can be ruled out. Pollen cones of Pinaceae [53] and Cupressaceae [54] bear peltate microsporophylls with free subpendulus microsporangia (pollen sacs) attached to the laminate heads of microsporophylls. The catkin-like pollen cones of Podocarpaceae are composed of peltate microsporophylls with two free microsporangia. In short, establishment of a new genus and species is justified by the distinctiveness of our material compared to pollen cone architecture of other extinct and extant gymnospermous groups.

#### In situ pollen and taxonomic affinities

From the holotype of the new taxon, we extracted in situ pollen that is assignable to the *Eucommiidites*-type, placing it within the Erdtmanithecales, a group known only from isolated reproductive organs, but whose affinities with other seed plants remains unclear [40]. The presence of dispersed *Eucommiidites* pollen in the mudstones of the Crato Formation, along with a diverse palynoflora including representatives of araucarian and cheirolepidiaceous conifers, gnetaleans, cycads, bennettiataleans, angiosperms, horsetails, ferns and freshwater algae was noted [19]. Unfortunately, the pollen sacs were not recovered, so we lack information on their placement, number, and morphology.

*Eucommiidites* pollen has been reported in situ in cones with peltate microsporophylls (*Erdtmanitheca texensis* from the Cretaceous of North America [55], *Erdtmanitheca portucalensis* from the Early Cretaceous of Portugal [56], *Eucommiitheca hirsuta* from the Early Cretaceous of Portugal [40, 56], and *Bayeritheca hughesii* from the Cenomanian of Bohemia in Central Europe [57]), and within *Erdtmanispermum* seeds [40, 55]. From this, Friis and Pedersen [40] established the Erdtmanithecales, an extinct gymnosperm group that is recognised solely from reproductive parts to date. Our taxon does not completely agree with the stalked peltate microsporangiate unit diagnosis, but the presence of the in situ *Eucommiidites* pollen indicates that the order is broader than originally thought. These other pollen cones assigned to the Erdtmanithecales are rather smaller and show a distinct phyllotaxis, opposite-decussate in the genus *Eucommiitheca* and radial or whorled in the genus *Erdtmanitheca* [40]. For *Bayeritheca*, the phyllotaxis is less clear [57]. In *Erdtmanitheca portucalensis*, the much smaller microsporophylls radiate from a central axis, although their barrel-shape with slightly depressed peltate head morphology is similar to our cones. Interestingly, the most complete specimen of this fossil species is a fragment that could superficially correspond to a hypothetical fragment of our detached cone portions. Mendes et al. [45] calculate the number of microsporophylls of a complete cone of *Erdtmanitheca portucalensis* as 100–150, which would correspond to the number of microsporophylls of our cone portions. However, microsporophylls of *Erdtmanitheca portucalensis* are less than 2 mm long.

*Araripestrobus* gen. nov. is also the first evidence of members of the Erdtmanithecales in the megafossil component of the Crato Formation and from South America. Megafossil remains of this group, namely microsporangiate cones and isolated seeds, were hitherto reported from the Early Cretaceous of North America and Europe. However, other plants parts and therefore plant habitus and size are, as yet, undiscovered. Recently, Friis et al. [10] hypothetically concluded, based on the sizes of mesofossils (isolated seeds) from their proposed BEG group (Bennettitales, Erdtmanithecales, Gnetales), that plants like *Drewria potomacensis* [58] could represent Erdtmanithecales. If so, the Crato plant *Cariria orbiculoconiformis*, which shows some remarkable similarities to *Drewria* [33], may also belong to this group. However, the reproductive organs are distinct and by far smaller than *Araripestrobus* gen. nov.

*Eucommiidites* pollen is ‘trisulcate’ with a broad distal sulcus and two narrower additional ‘sulci’ about 120° away from the sulcus [59], here termed pseudosulci. In the original descriptions, the terms colpus and pseudocolpus have been used, but we update the terminology to sulcus and pseudosulcus as this is correct when dealing with gymnospermous pollen. A sulcus is an elongated aperture distally located, whereas a colpus is an equatorial elongated aperture and in some descriptions the main ‘colpus’ is described as distal. In our new species, the pollen has a sulcus membrane with clearly rounded ends. In *Araripestrobus resinosus* gen. nov. et sp. nov. the pollen is finely perforate varying from smooth to almost microrugulate between the perforations, occasionally micro-fossulae are seen. The variation in the pollen surface and its size make it different to all other *Eucommiidites* pollen species described so far (see [60]).

Palynological studies of the Araripe Basin recorded dispersed pollen grains assigned to *Eucommiidites troedssonii* and *E. minor*, plus three other unnamed *Eucommiidites* species [61]. However, the samples are thought to lack firm stratigraphic control, so the precise lithostratigraphic ages are not known and may include material that is older or younger than the Crato Formation flora [19]. However, the in situ pollen is most similar in size to the recovered *E. troedssonii*, but differs its ornamentation, whereas *E. minor* pollen is smaller and laevigate [62]. The in situ pollen is also much larger than the three unnamed *Eucommiidites* species described; additionally, of these unnamed species, two are psilate and the third is lightly scabrate but almost spherical in outline [61].

The in situ pollen recovered is most similar in length to, although slightly larger than, in situ pollen of *Erdtmanitheca portucalensis*, but in width to in situ pollen found inside *Erdtmanitheca texensis*. In situ pollen of *Erdtmanitheca texensis* measures 20–25 μm long and 15–19 μm wide, and has lateral pseudosulci that are shorter than the main sulcus. The pollen surface is mainly psilate but with a few prominent granules and associated pits [55]. In situ pollen of *Erdtmanitheca portucalensis* measures 16.0–27.2 μm × 11.9–16.4 μm and has a well-defined sulcus, with slightly expanded rounded ends. Two slit-like lateral pseudosulci, longer than the sulcus, are present more or less in the equatorial plane. Margins of the apertures are irregular and the aperture membrane is verrucate-granular. The pollen wall is psilate, although puncta are seen in some specimens [45]. The pollen inside *Eucommiitheca hirsuta* is elliptical in equatorial outline, about 15–20 μm long and 10–12 μm wide, typically pointed or truncate at the ends, with three parallel and almost equal length sulci, and a smooth and faintly foveolate pollen wall [40]. *Eucommiidites* pollen discovered inside *Erdtmanispermum balticum* seeds is distinctly foveolate and much smaller (22 μm by 15 μm, [55, 60]) than our larger and finely perforate in situ pollen.

*Bayeritheca hughesii* is a single elongate cone that has in situ *Eucommiidites* pollen in synangia, not separate sporangia, unlike *Erdtmanitheca* and *Eucommiitheca* [57]. The pollen has one normally straight sulcus that does not reach the equator, commonly with rounded ends. The two pseudosulci are usually curved, occasionally joined at one end to form a single ring furrow, unlike other in situ *Eucommiidites* pollen, including that reported here. The distal half of the grains is more flattened than the proximal half, which is convex. The pollen grains are psilate in light microscopy, and finely granulate with protruding globular elements [0.1–(0.3) 0.4 μm] under SEM. The pollen grains are 14–16 (15.2) μm long; 10–13 (11.2) μm wide, smaller than the pollen reported here. Interestingly, the macerated microsporangiate units have small resin bodies (40–65 μm in diameter) present [57]. *Bayeritheca hughesii* is, so far, the largest male reproductive structure described from the Erdtmanithecales but even these cones are conspicuously smaller than *Araripestrobus* gen. nov.

Pollen grains resembling *Eucommiidites* were also found in situ in cones of *Hastyostrobus muirii* from the Middle Jurassic of Yorkshire, England [63]. Based on the wall ultrastructure, Tekleva et al. [64] believe that the pollen is assignable to *Eucommiidites*, but this was doubted by Mendes et al. [45]. Resinous microsporophylls of the early Cretaceous *Loricanthus resinifer*, initially appear to resemble one of our specimens (Fig. 2f), but they are peltate, borne in smaller strobili and have a more rounded (not *Eucommiidites*–type) pollen grain in situ, so the affinity of the *Loricanthus* plant is unknown [65, 66].

Our in situ pollen cannot be assigned to any currently described *Eucommiidites* species. We refrain from giving our in situ pollen a name as the cones themselves are named. It is interesting to note that although we have pollen assignable to *Eucommiidites*, the reproductive cone structure is tremendously different from all other structures bearing *Eucommiidites*-type pollen, in the layered appearance and helical arrangement of the non-peltate microsporophylls. Additionally, the new taxon exhibits prominent resin canals, a feature not described for other members of the Erdtmanithecales. This unusual feature, along with the differences in cone morphology may either indicate that the poorly known Erdtmanithecales encompasses more diversity than originally thought, or that more than one plant group produced *Eucommiidites*-type pollen. If the habitus of Erdtmanithecales is putatively shrub, sub-shrub or herbaceous-like is assumed by [10] the latter might be the case because the large *Araripestrobus* gen. nov. cones likely hint towards larger plants.

### Amber classification

#### FTIR spectra of ambers

Amber from *Araripestrobus* gen. nov. is similar to the *Brachyphyllum* amber measured here in parts of its general spectrum, but a key difference is the lack of a strong peak at 1690 cm^− 1^, and the presence of a peak at 1645 cm^− 1^, which appears as a shoulder to the large peak at 1690 cm^− 1^ present in *Brachyphyllum*. This suggests that there is a slight difference in the amber chemistry, but it is not clear if this has resulted from differences in the original resin chemistries between the plants, or differences in maturation or weathering. Maturational differences cannot be excluded since all the plants are from the C6 limestone of the Crato Formation, but perhaps from different horizons that are distinguished by colour, grain size and other macroscopic rock features. Recent publications on the Crato Formation reinforce these differences are likely caused by fluctuations in water depth and water chemistry [67–69]. Weathering differences are a possibility too since different quarries at different locations in the Araripe Basin operate to retrieve the limestones, and different specimens may have been also exposed after collection for different durations. The process of resin maturation [70], also named amberization, causes changes in the chemical composition of fossil resins, and the rate of this phenomenon depends on several variables, such as temperature, age and thermal history of the sample. Resins with similar palaeobotanical origin may present different compositions, as indicated by infrared spectroscopy, in consequence of several taphonomic variables. These results from FTIR spectrum can be further confirmed by Pyr-GC-MS analysis which can give a more specific assignment of chemical structures of the fossil resin (see below).

The in situ Crato Formation amber spectrum, with a biphasic weak absorption band between 1240 cm^− 1^ and 1015 cm^− 1^ is also partially similar to that of New Zealand amber (thought to originate from the araucarian genus *Agathis,* see [71]), and of extant kauri (*Agathis australis*) resin [2, 71–73]. Conversely, the fingerprint region appears different from that of present-day *Pinus* resin [72, 73].

Further comparisons can be done with spectra from other fossil resins, considering the fingerprint region, where absorption caused by carbon-oxygen single bonds, as well as aromatic ethers and phenols occurs. This region from the in situ Crato Formation amber spectrum shows a pattern similar to that of Cedar Lake (Manitoba, Canada) Cretaceous amber and also to that of Atlantic Coastal Plain fossil resin ([2]; page 89 and plates XIV, XVI), attributed to family of Araucariaceae. However, recent FTIR analyses of the Grassy Lake ambers, along with fossil plant fragments found within the ambers, showed a closer relationship to Cupressaceae [74]. The Atlantic Coastal Plain or Raritan (New Jersey) amber originated from the Cupressaceae [75], with additional chemical evidence provided using Pyr-GC-MS [76].

Our amber spectra appear to have strong affinities with those of other Cretaceous presumed araucariacean-derived ambers, including Cretaceous ambers from Spain [77], and Cretaceous ambers from France [78]. Infrared (IR) analyses of amber have been previously interpreted to give differing results in some cases; however, Tappert et al. [73], using micro-FTIR, showed that conifer resins can effectively be classified into two groups: ‘pinaceous resins’ from Pinaceae, which consist mainly of abietane/pimarane diterpenes, and ‘cupressaceous resins’, which they associate with Cupressaceae, Sciadopityaceae, Araucariaceae, and Podocarpaceae, and consist mainly of labdanoid diterpenes. Our ambers would fall in to ‘cupressaceous resins’ group identified by Tappert et al. [73], which may have an araucariacean or cupressacean, but not a pinaceous affinity. Notably, no ambers from other gymnosperm groups were known until now, so this grouping may have to be expanded to include fossil resins from these enigmatic erdtmanithecalean plants.

### Pyrolysis gas chromatography mass spectroscopy

The in situ amber belongs to the class Ib group of resins, which is the most common form of amber in the geosphere. Class Ib ambers are formed by copolymers of regular labdanoid diterpenes, especially communic acid, communol and biformenes. In this case, the distribution of products observed indicates that the macromolecular structure of the resin is dominated by communic acid (products from communal and biformene are essentially absent). No occluded intact diterpenes are observed, therefore, an affinity to the Pinaceae can be excluded as their resins are rich mostly in abietane and pimarane diterpenic acids and lack significant amounts of free or macromolecular labdanoids (see also [73, 79]). We cannot exclude an affinity with the Araucariaceae or Cupressaceae, which are rich in labdanes, including communic acid and agathic acid [76, 80], nor the Podocarpaceae, and Taxodiaceae. There are minor amounts of fatty acids, but no biomarkers present meaning that a closer identification of the source plant is not possible. The absence of intact occluded diterpenes and the high ratio of C_15_/C_14_ bicylic products observed in the pyrolysate derived from this amber suggests that it has experienced a moderate degree of thermal stress during its history.

The assignment of the amber to class Ib excludes an affinity with the highly resinous angiosperm *Hymenaea*, which forms class Ic ambers, which are linked to the relatively common, but much younger Dominican and Mexican ambers. Class Ia ambers are Paleogene Baltic ambers which have succinic acid present [80], which is clearly absent from the amber analysed here. What is interesting is that the amber has as similarity with a Mesozoic amber found in archaeological context in South America [81], but for which the palaeobotanical affinities are unknown.

Taken together, the FTIR analysis and the Pyr-GC-MC indicate that there is no clear way to pinpoint to which botanical group the resin from *Araripestrobus* gen. nov. can be chemically placed, only which groups (Pinaceae, *Hymenaea*) can be excluded. The presence of the in situ pollen showing an erdtmanithecalean affinity, suggests that other gymnosperm source plants of amber have not been detected until now.

## Conclusions

We used multiple lines of evidence to uncover the botanical affinity of cones and detached cone portions with in situ amber and in situ pollen, and to understand the potential amber sources of the Crato Formation flora, beyond the araucariacean source plants already known. The in situ pollen shows that *Araripestrobus resinosus* gen. nov. et sp. nov. is a microsporangiate organ of gymnospermous affinity, displaying a very dissimilar macromorphology to any pollen cones of extinct and extant taxa described to date. Based on the presence of in situ *Eucommiidites*-type pollen, the new genus and species is placed in the order Erdtmanithecales, but based on differences in the cone morphologies they cannot be placed within the only hitherto known family, Erdtmanithecaceae. Besides dispersed pollen, these specimens represent the first macrofossil evidence of the Erdtmanithecales in South America. The FTIR analyses of the in situ amber implies an affinity with some conifers (araucariacean or cupressacean), by effectively ruling out an affinity with the Pinaceae. The Pyr-GC-MS analysis shows this amber to be a class Ib resin that has undergone some thermal maturation. From this, it is clear that the new erdtmanithecalean taxon produced a resin that has matured to an amber very similar to the labdane-based resins and ambers thought to derive from some conifer groups, particularly araucariaceans, cupressaceans, and cheirolepidiaceous conifers, but clearly excludes a pinaceous affinity.

## Data Availability

All specimens are available from their listed repositories: Museum für Naturkunde Berlin, University of Portsmouth Collection, Senckenberg Research Institute and Natural History Museum Frankfurt/M., Universidade Estado de Rio de Janeiro, and Fundação Paleontológica Phoenix, Ceará, Brazil.

## Abbreviations

BEG group: Bennettitales-Erdtmanithecales-Gnetales group
FTIR: Fourier-transform infrared
IR: Infrared
LM: Light microscopy
Pyr-GC-MS: Pyrolysis gas chromatography mass spectroscopy
SEM: Scanning electron microscopy
